# Effects of Diet Macronutrient Composition on Weight Loss during Caloric Restriction and Subsequent Weight Regain during Refeeding in Aging Mice

**DOI:** 10.3390/nu15224836

**Published:** 2023-11-19

**Authors:** Petras Minderis, Andrej Fokin, Tomas Povilonis, Mindaugas Kvedaras, Aivaras Ratkevicius

**Affiliations:** 1 Institute of Sport Science and Innovations, Lithuanian Sports University, 44221 Kaunas, Lithuania; andrej.fokin@lsu.lt (A.F.);; 2 Faculty of Medicine, Lithuanian University of Health Sciences, 44307 Kaunas, Lithuania; 3 Department of Health Promotion and Rehabilitation, Lithuanian Sports University, 44221 Kaunas, Lithuania; aivaras.ratkevicius@lsu.lt; 4 Sports and Exercise Medicine Centre, Queen Mary University of London, London E1 4NS, UK

**Keywords:** diets, fat loss, weight cycling

## Abstract

Caloric restriction (CR) induces weight loss, but is associated with rapid weight regain upon return to ad libitum feeding. Our aim was to investigate effects of the macronutrient composition of the diet on weight loss and regain in elderly mice. Males, 18 months old, of the C57BL/6J strain were subjected to 4-week 30% CR followed by 4 weeks of ad libitum refeeding on either high-carb (HC), high-fat (HF) or high-protein (HP) diets (*n* = 22 each). Mice (*n* = 11) fed a chow diet ad libitum served as a control group (CON). Body mass and food intake were monitored daily. Twenty-four-hour indirect calorimetry was used to assess energy expenditure and substrate oxidation. Muscle and fat mass were evaluated with dissection of the tissues. Serum leptin and ghrelin levels were also measured. CR-induced weight loss did not differ between the diets. Weight regain was particularly fast for HF as mice overshot their initial weight by 12.8 ± 5.7% after 4-week refeeding when HC and HP mice reached the weight of the CON group. Weight regain strongly correlated with energy intake across the groups. The respiratory exchange ratio was lower in HF mice (0.81 ± 0.03) compared to HC (0.94 ± 0.06, *p* < 0.001), HP (0.89 ± 0.04, *p* < 0.001) and CON mice (0.91 ± 0.06, *p* < 0.01) during the refeeding. Serum leptin levels were higher in HF mice (1.03 ± 0.50 ng/mL) compared to HC (0.46 ± 0.14, *p* < 0.001), HP (0.63 ± 0.28, *p* < 0.05) or CON mice (0.41 ± 0.14, *p* < 0.001). Thus, CR induces similar weight loss in aging mice irrespective of the diet’s macronutrient composition. An HF diet leads to excessive energy intake and pronounced gain in body fat in spite of increased fat oxidation and serum leptin during the refeeding after CR.

## 1. Introduction

Excessive energy intake leading to energy imbalance is recognized as one of the major causes of obesity as shown in both human and animal studies [1,2]. Compared to their ancestors, modern humans consume more calories and engage in less physical activity [3]. Physical activity might be used as a tool in weight management, but recent studies suggest that it has a limited impact on the overall energy expenditure due to compensatory behavior and/or likely changes in movement economy [4,5]. It appears that human energy expenditure operates within a rather narrow range and is paradoxically similar between contemporary Western and hunter–gatherer societies, despite significant differences in activity levels [6]. GIP and GLP-1 receptor agonists, which are effective in inducing weight loss, operate by reducing energy intake rather than expenditure [7,8]. Therefore, while physical activity plays a vital role in overall health and contributes to weight maintenance, targeting appetite regulation and energy intake remains a critical aspect of interventions to reduce obesity.

Caloric restriction (CR) is an effective intervention for weight loss regardless of the diets or dietary macronutrient allocation followed [9,10,11]. However, weight loss and related health benefits often disappear after return to the unrestricted feeding [12]. A better understanding of the mechanisms driving this weight rebound might help in development of new sustainable strategies for weight management. Effects of dietary composition on this weight rebound remain poorly understood. Studies of C57BL/6 mice are well suited to address this lack of knowledge as these mice are prone to obesity and show similar metabolic dysfunctions as humans [13].

Along with increased life expectancy and food availability, obesity is becoming increasingly prevalent in the elderly [14]. The aim of our current study was to investigate effects of dietary macronutrient composition on physiological adaptations of old C57BL/6 mice to 30% CR followed by a period of ad libitum feeding. Our previous study showed that weight and fat loss does not depend on macronutrient proportions of the diet in adult mice undergoing CR [11]. It is important to investigate if this is true for old mice, which show greater weight loss than adult mice when subjected to 30% CR [15]. There is evidence that a high-protein diet might be effective in reducing the body fat increase during the refeeding after CR [16,17]. Furthermore, the ratio of carbohydrates to fat in an unrestricted diet appears to have varying effects on gut-derived appetite hormones and insulin, which might influence body fat regain during the refeeding phase [18,19]. However, food intake is subject to significant variations and difficulties in assessment in free-living humans. Therefore, studies of genetically homogeneous C57BL/6 mice that compare diets under well-defined conditions provide a useful test for conclusions drawn from human studies.

## 2. Materials and Methods

### 2.1. Mice and Experimental Design

The study was carried out at the animal research facility of the Lithuanian Sports University with approval of all the procedures by the Lithuanian State Food and Veterinary Service (ref. #G2-90). The C57BL/6J mice were obtained from the Jackson laboratory (Bar Harbor, ME, USA) and housed at the ambient temperature of 20–21 °C, 40–60% humidity, with a 12 h light/dark cycle. After weaning, male mice were selected for the study and kept up to five littermates per cage, fed a standard chow diet (1320 M, 24% kcal protein, 15% kcal fat, 61% kcal carbohydrates; Altromin, Lage, Germany) ad libitum and received unrestricted access to tap water. Mice were kept under these conditions until 17 months of age, and then placed into separate cages. During the 18th month, the baseline food intake of each mouse was evaluated over the period of 3 weeks. This was performed by subtracting the weight of food leftovers at the end of each week from the initially provided food weight with corrections for a humidity effect on the pellets’ weight.

### 2.2. Caloric Restriction and Refeeding

Initially, 18-month-old C57BL/6J mice were subjected to 30% CR for 4 weeks on (1) high-fat (HF; 60%, 20% and 20% kcal from fat, carbohydrate and protein, respectively), (2) high-carbohydrate (HC; 20%, 60%, 20% kcal from fat, carbohydrate and protein, respectively) or (3) high-protein (HP; 30%, 35% and 35% kcal for fat, carbohydrate and protein, respectively) diets. Each group consisted of 22–23 animals, and received purified diets (Ref# D12451 for HF, Ref# D17100401 for HC, Ref# D17100402 for HP, respectively) produced by Research Diets Inc. (New Brunswick, NJ, USA). Additional information about the diets is presented in our previous study [11]. The amount of food provided was adjusted for the different energy densities of the diets (5.2, 4.1 and 4.3 kcal/g for HF, HC and HP, respectively) to match the total energy content in the groups. Mice were fed daily at 9–10 a.m. After 4-week CR, mice were allowed to eat unrestrictedly for another 4 weeks using the same diets. Energy intake was calculated daily by weighing food leftovers adjusted for the energy density of the diet. Eleven mice served as controls (CON) and were fed a standard chow diet ad libitum throughout the whole course of the study.

### 2.3. Metabolic Measurements

During the final week of both CR and refeeding (post-caloric restriction, P-CR), 24 h energy expenditure and physical activity were assessed. Each mouse was weighed and transferred to the metabolic cage with restricted or ad libitum access to food according to the study phase (CR or refeeding). The measurements were started at 9 a.m. and continued until 9 a.m. of the following day to cover the light (from 9 a.m. to 9 p.m.) and dark (from 9 p.m. to 9 a.m.) periods of the day. The metabolic cage was connected to a gas analyzer (LE405, Panlab Harvard Apparatus, Barcelona, Spain) and a switching device (LE400, Panlab Harvard Apparatus, Barcelona, Spain) to control the air flow. The gas analyzer was calibrated at the high point (50% O_2_, 1.5% CO_2_) and low point (20% O_2_, 0% CO_2_). Air flow was set at 250 mL/min, with a 3 min switching time between measurements of O_2_ and CO_2_ concentrations inside and outside the metabolic cage. The respiratory exchange ratio (RER) and energy expenditure were calculated using Metabolism software version 1.2 (Panlab Harvard Apparatus, Barcelona, Spain). Physical activity was assessed using strain gauges mounted on the supporting constructions of the metabolic cage, and the integral of ground reaction forces was used as an indirect measure of physical activity.

### 2.4. Body Mass and Composition

Body mass of mice was monitored daily using scales with a precision of 0.1 g (440–45 N, Kern, Balingen, Germany). Half of the mice (*n* = 10–11 in each group) were euthanized midway through the study using inhalation of CO_2_ for assessment of changes in body composition after CR. The remaining mice (*n* = 11–12 in each group) were euthanized at the end of the subsequent refeeding period. Hindlimb muscles (including gastrocnemius, plantaris, soleus, tibialis anterior and extensor digitorum longus) were dissected bilaterally, and the summed weight of all the muscles served as a muscle mass index. Muscles were trimmed from all visible tendons and blotted dry before being weighed on analytical balances with a precision of 0.1 mg (ABS 80-4, Kern, Balingen, Germany). White adipose tissue (subcutaneous, gonadal, mesenteric and perirenal fats) and brown adipose tissue (intrascapular fat) were dissected to assess changes in body fat [11]. The summed weight of all fat served as a body fat index.

### 2.5. Serum Leptin and Ghrelin

Blood samples from mice were collected after a 6–8 h fasting using heart puncture immediately following sacrifice. Serum was separated with a 2-round centrifugation at 4 °C and 4000 rfc for 15 min and at 4 °C and 4000 rfc for 2 min, frozen and stored at −80 °C for a later analysis. Serum concentrations of leptin (RAB0334, Sigma-Aldrich, St. Louis, MO, USA) and ghrelin (E-EL-M0551, Elabscience, Houston, TX, USA) were determined using the ELISA protocol for a fluorescent colorimetric analysis with a plate reader (Spark 10 M, Tecan Group Ltd., Männedorf, Switzerland). Samples were analyzed in duplicates to ensure accuracy and reported as average values.

### 2.6. Statistical Analysis

Data were analyzed using GraphPad Prism 6.0 software. Data normality was verified with the Shapiro–Wilk test. Two-way ANOVA was performed to determine the effects of the dietary phase (CR and refeeding), diet (HC, HF and HP) and dietary phase * diet interaction. Two-way repeated measures (RM) ANOVA was used to investigate the effects of the diet, time (hours or days of intervention) and diet * time interaction in the same mice. One-way ANOVA was used to compare the CON group with other diet groups at a specific dietary phase (CR or refeeding). Bonferroni post hoc tests were used to evaluate mean differences between groups. The Kruskal–Wallis test with a Dunn post hoc analysis was used when the data were not normally distributed. A linear regression analysis was performed on the plots of changes in body mass over energy intake during refeeding. The Pearson correlation coefficient was calculated to assess the strength of the association. *p* < 0.05 was considered significant.

## 3. Results

### 3.1. Body Mass

**Figure 1:**
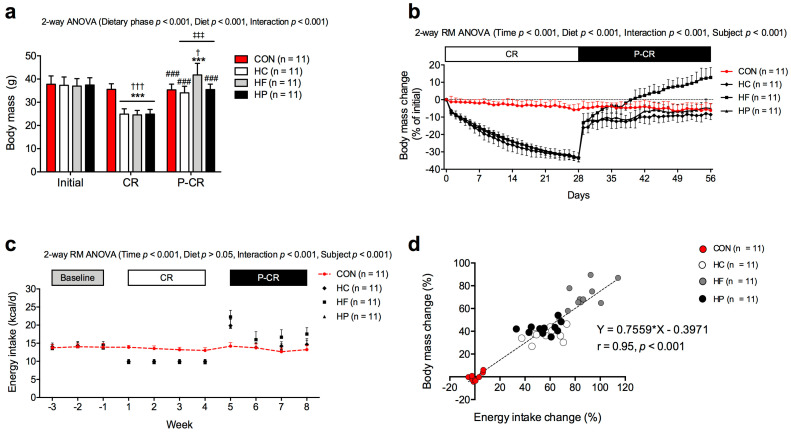
Changes in body mass and energy intake for the control group (CON; red color) as well as for mice subjected to 4-week caloric restriction (CR) followed by 4-week post weight loss ad libitum refeeding (P-CR) on high-carbohydrate (HC), high-fat (HF) or high-protein (HP) diet. Data on body mass are shown in (**a**) absolute values and (**b**) daily percentage change from initial values, and data on energy intake are shown as (**c**) weekly change and (**d**) as a relationship between percentage increase in energy intake and body mass regained during refeeding. Data are expressed as mean ± SD (in panel (**a**–**c**)). In panel (**a**), two-way ANOVA with Bonferroni post hoc analysis was performed to assess effects of dietary phase; diet; and phase and diet interaction. In panel (**b**,**c**), two-way repeated measure (RM) ANOVA with Bonferroni post hoc analysis was performed for effects of time, diet, time and diet interaction and individual subject. *** *p* < 0.001 vs. CON; † *p* < 0.05, ††† *p* < 0.001 vs. initial, ‡‡‡ *p* < 0.001 vs. CR; ### *p* < 0.001 vs. HF. In panel (d), Pearson correlation coefficient (r) and linear regression equation for the plots of body mass change versus energy intake change are shown with each dot representing one mouse data sample.

Figure 1 illustrates changes in body mass and energy intake of mice during CR and refeeding (P-CR). All groups had a similar body mass at the baseline (Figure 1a). CR induced a similar weight loss for all diets when compared to the baseline or CON group (*p* < 0.001). Return to ad libitum feeding led to weight regain. HF mice showed markedly greater weight increases than the other diet groups (*p* < 0.001).

As shown in Figure 1b, the diet groups experienced a similar rate of decline as the relative weight loss was similar in HF (−33.6 ± 2.1%), HC (−33.2 ± 2.6%) and HP (−33.4 ± 2.8%) groups after CR. Upon refeeding, weight regain was particularly fast during the first day. Afterwards, HC and HP groups stabilized their weight regain at a slower rate. The HF group showed a significantly faster weight regain than HC and HP groups, leading to a 12.8 ± 5.7% weight overshoot after refeeding compared to the baseline. Body mass of the HF group was greater than in HC and HP groups (*p* < 0.001) already on the 5th day of refeeding and remained so until the end of the refeeding. HC and HP groups reached a similar weight as the CON group, which showed a slight weight loss (6.2 ± 4.0%).

Data on the energy intake during the experiment are shown in Figure 1c. Energy intake of the CON group remained similar throughout the experiment. After a deliberate 30% caloric restriction, the diet groups significantly increased their energy intake during the refeeding (*p* < 0.001), especially during the first week, when allowed to eat ad libitum. During the entire refeeding period, the HF group consumed 87 ± 14% more food energy than during the previous period of caloric restriction of the same duration, and this increase was significantly greater compared to the other diet groups (HC and HP increases were 57 ± 11% and 55 ± 12%, respectively; *p* < 0.001). Association between energy intake and weight gain during the refeeding is shown in Figure 1d. There was a strong positive correlation between changes in energy intake and body mass (r = 0.95, *p* < 0.001). The CON group maintained a consistent energy intake throughout the study and their body mass remained relatively stable. Mice from all three diet groups showed significant increases in energy intake during the refeeding. There was a clear difference in this response for the HF group compared to the other diet groups, as mice in this group almost doubled their energy intake.

### 3.2. Body Composition

**Figure 2:**
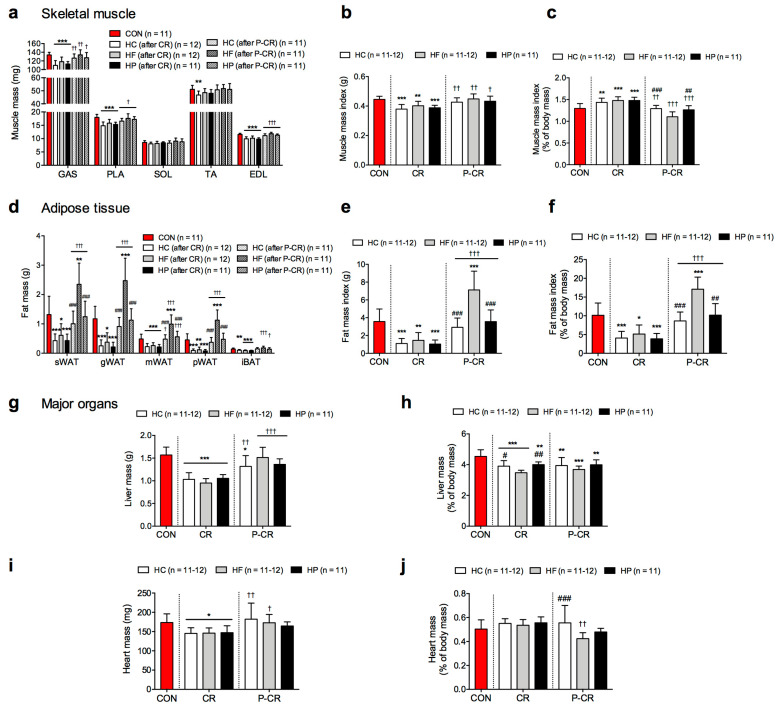
Weights of dissected mouse tissues and organs, including hindlimb muscles (**a**–**c**), fats from different sampling sites (**d**–**f**), liver (**g**,**h**) and heart (**i**,**j**), after a 4-week caloric restriction (CR) followed by 4-week post weight loss ad libitum refeeding (P-CR) with diets of different macronutrient composition (high-carbohydrate, HC; high-fat, HF; high-protein, HP) compared to control group (CON; red color) (*n* = 11–12 each). Data are expressed as mean ± SD. One-way ANOVA with Bonferroni post hoc analysis was performed to compare CON group with diet groups at particular nutritional phase. Two-way ANOVA with Bonferroni post hoc analysis was performed for effects of dietary phase, diet, phase and diet interaction (**a**–**c**,**g**–**j**). Kruskal–Wallis test with Dunn post hoc analysis was used when the data were not normally distributed (**d**–**f**). * *p* < 0.05, ** *p* < 0.01, *** *p* < 0.001 vs. CON; † *p* < 0.05, †† *p* < 0.01, ††† *p* < 0.001 vs. CR; # *p* < 0.05, ## *p* < 0.01, ### *p* < 0.001 vs. HF at same phase (CR or P-CR). Tissue abbreviations: GAS, gastrocnemius; PLA, plantaris; SOL, soleus; TA, tibialis anterior; EDL, extensor digitorum longus; sWAT, subcutaneous white adipose tissue; gWAT, gonadal white adipose tissue; mWAT, mesenteric white adipose tissue; pWAT, perirenal white adipose tissue; iBAT, intrascapular brown adipose tissue.

Figure 2 presents data on changes in body composition. We euthanized half of the animals in order to assess effects of CR on body composition in the diet groups. Three out of five hindlimb muscles (gastrocnemius, plantaris and EDL) were consistently smaller in the diet groups after exposure to CR compared to the CON group (*p* < 0.001) (Figure 2a). Soleus muscle weight did not differ from the CON group. Tibialis anterior weight decreased (*p* < 0.01) only after HC. After CR, the absolute muscle mass index was reduced (Figure 2b) while the relative muscle mass index was increased (Figure 2c) in the diet groups compared to the CON group. All the diet groups regained their absolute muscle mass to the level of the CON group after refeeding (Figure 2b), but the relative muscle mass index of the HF group was significantly lower (*p* < 0.01) after refeeding compared to the other diet and CON group (Figure 2c).

CR induced a decrease in fat at all sampling sites (Figure 2d), and the most significant change occurred in HC and HP groups (*p* < 0.001). Refeeding led to recovery of body fat as HC and HP groups restored their percentage of body fat to the level of the CON while the HF group exceeded the CON group, increasing their body fat percentage almost two-fold (17% vs. 10%, *p* < 0.01) (Figure 2f).

Liver mass was nearly 30% lower after CR than in the CON group (*p* < 0.001) (Figure 2g). Even when normalized to body mass, liver mass was reduced in diet groups after CR compared to the CON group (Figure 2h). Refeeding lead to significant recovery of liver weight, but its relative weight remained lower compared to CON (*p* < 0.01) in all diet groups. Heart mass was also reduced after CR compared to CON (*p* < 0.05), but the reduction was not as notable as for the liver (Figure 2i). The relative heart mass remained similar in all dietary phases in HC and HP groups, but not the HF group, which showed a reduction after refeeding (Figure 2j).

### 3.3. Serum Ghrelin and Leptin

**Figure 3:**
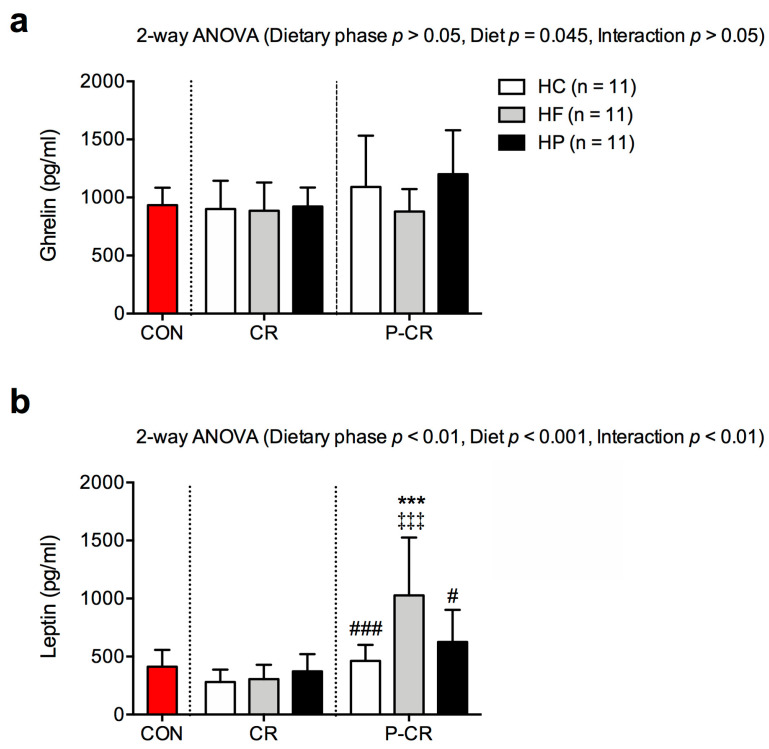
Serum ghrelin (**a**) and leptin (**b**) concentrations in mice from the control group (CON; red color) and mice on high-carbohydrate (HC), high-fat (HF), high-protein (HP) diets subjected to 4-week caloric restriction (CR) followed by 4-week refeeding (P-CR). Data are expressed as mean ± SD. One-way ANOVA with Bonferroni post hoc analysis was performed to compare CON group with diet groups at particular nutritional phase. Two-way ANOVA with Bonferroni post hoc analysis was performed for effects of dietary phase, diet, phase and diet interaction. *** *p* < 0.001 vs. CON; ‡‡‡ *p* < 0.001 vs. CR; # *p* < 0.05, ### *p* < 0.001 vs. HF.

Data on serum ghrelin and leptin concentrations are presented in Figure 3. There were no significant differences in ghrelin concentrations between the studied groups of mice (Figure 3a). Leptin concentrations did not differ between the groups after CR either. However, after refeeding, the HF group had significantly higher leptin concentrations than the other groups (Figure 3b).

### 3.4. Energy Metabolism

**Figure 4:**
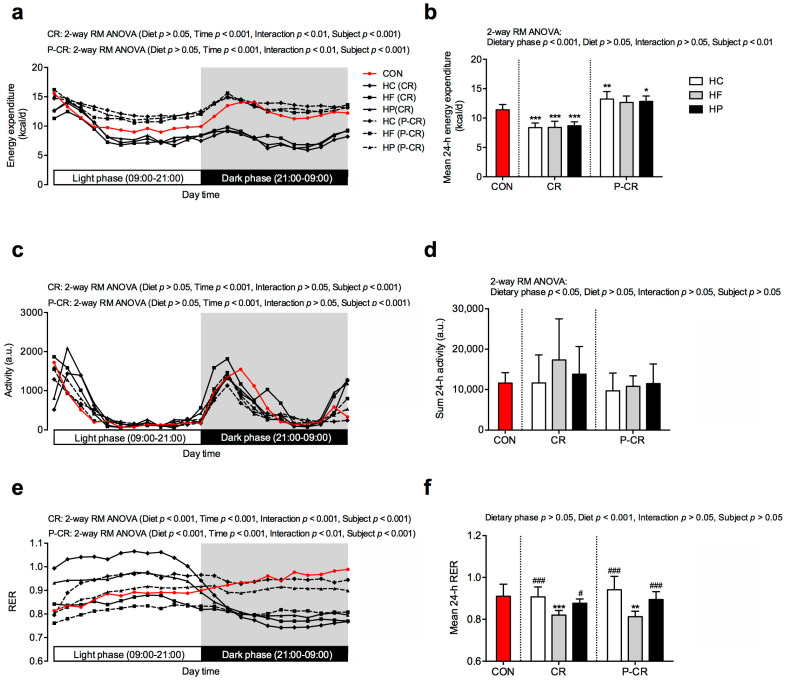
Twenty-four-hour energy expenditure (**a**,**b**), locomotory activity (**c**,**d**) and respiratory exchange ratio (RER) (**e**,**f**) in mice from the control group (CON; red color) and mice on high-carbohydrate (HC), high-fat (HF), high-protein (HP) diets subjected to 4-week caloric restriction (CR) followed by 4-week refeeding (P-CR). Data are expressed as mean ± SD. Two-way repeated measure (RM) ANOVA with Bonferroni post hoc analysis was performed for effects of diet, time (**a**,**c**,**e**) or dietary phase (**b**,**d**,**f**), interaction (diet and time or diet and dietary phase) and subject. One-way ANOVA with Bonferroni post hoc analysis was performed to compare CON group with diet groups at particular dietary phase. * *p* < 0.05, ** *p* < 0.01, *** *p* < 0.001 vs. CON; # *p* < 0.05, ### *p* < 0.001 vs. HF.

As shown in Figure 4, average 24 h energy expenditure was lower (*p* < 0.001) in mice subjected to CR compared to CON and then increased (*p* < 0.001) after refeeding (P-CR) without a significant difference between the diets. Differences in energy expenditure between CR and P-CR phases were particularly pronounced during the dark phase, when mice are typically active (Figure 4a). For the CR phase, diet groups had lower energy expenditure than the CON group at almost all light and dark phase time points (Figure 4a). Under the P-CR conditions, diet groups showed greater energy expenditure in the light phase but not in the dark phase compared to the CON group.

Data on locomotor activity of mice are presented in Figure 4c,d. Activity levels varied considerably between animals without significant differences between the diet groups. Mice were more active during CR than the refeeding phase (*p* < 0.05) (Figure 4d). For the CR phase, mice were particularly active at the end of the dark phase, just before the daily feeding at 9–10 a.m. (Figure 4c).

Data on the respiratory exchange ratio (RER) are presented in Figure 4e,f. The diet had a significant (*p* < 0.001) effect on the values. For both CR and P-CR, the HF group showed lower RER than the other groups. This difference was particularly noticeable in the light phase of CR as the diet groups ranked as follows from the highest to the lowest values: HC, HP and HF (Figure 4e). All the differences between HC, HP and HF disappeared in the dark phase of CR. Under P-CR conditions, the HF group had lower RER values than the other diet groups at all time points of the day.

## 4. Discussion

The main aim of our study was to investigate effects of different macronutrient proportions in the diet on changes in metabolism and body composition during CR and subsequent refeeding in aged mice. Our results show that these effects mostly depend on energy status. There were no significant differences between the diets applied in body weight or tissue loss during CR. However, the fastest weight and fat regain was observed in mice refed using a high-fat diet. This was observed in spite of elevated fat oxidation in these mice, as weight regain correlated with energy intake. Our results suggest that weight and fat gain is primarily dependent on the energy intake, which is linked to energy density of the food rather than ability to oxidize fat during refeeding.

The results of this study are consistent with our earlier findings on adult mice [11], where we observed similar changes in body composition of 6-month-old mice fed different diets during CR. In relation to human and mice lifespans, 6-month-old mice correspond to a human age of 20–30 years [20]. The current study was carried out on 18-month-old mice, which is equivalent to 60 years of age in humans. Health problems related to dietary factors have significant impact on health of ageing humans and it is important to verify findings on young mice using older mice [21].

Isolated muscle and fat mass indices showed that all diets had a similar effect on body composition of the aged mice exposed to CR. Body weight decreased mainly due to the loss of body fat. The skeletal muscles and heart that make up a significant proportion of lean body mass were more or less protected from this catabolic stimulus. However, similarly to young mice, the liver of aged mice showed a significant weight loss after CR [11,22]. It appears that CR induces functional and structural cellular remodeling in the liver, with glycogen depletion and/or alterations in other constituents having a significant impact on liver mass during fasting in mice [23]. Small and hierarchical changes in vital organs were also found in studies of graded levels of caloric restriction, from 10 to 40%, in mice [24]. In these studies, the contribution of body fat utilization was the most pronounced of all tissues and reached 55–60% by the end of CR. In all diet groups, perirenal and gonadal fat exhibited the most substantial relative decrease during caloric CR. The high-fat diet-induced regain of fat mass during refeeding was also comparatively the highest in these two fat depots. Perirenal adiposity, identified as a prognostic marker, has been associated with chronic kidney disease (CKD), cardiovascular disease (CVD) and metabolic syndrome [25,26]. These findings offer potential insights for the development of targeted nutritional interventions aimed at reducing the prevalence and risks of these interconnected conditions.

We anticipated that age may impact the response to the high-carbohydrate diet due to its association with ageing effects on metabolism [27]. A heated scientific debate concerns the role of carbohydrates in obesity pathogenesis [28,29]. According to some researchers, dietary carbohydrates, but not fat, promote weight gain by inhibiting lipolysis through insulin and decreasing energy expenditure, leading to increased adiposity [30]. However, mouse and human studies challenge this hypothesis when food is directly provided to subjects and rigorously controlled during CR [10,31]. It might be argued that, if such a mechanism of carbohydrate fattening were to exist, it would likely be more evident in conditions of unrestricted energy intake with significantly higher insulin secretion. An RCT study conducted with proponents of the carbohydrate–insulin model (CIM) demonstrated that reducing dietary carbohydrates increased energy expenditure during weight loss maintenance [18]. However, an absence of data on body weight and composition is a major limitation of this study, thereby hindering assessment of practical effects of such diets on weight control. Our mouse study found no differences between high- and low-carbohydrate diets when protein was equated in energy expenditure or activity levels during the refeeding phase. Furthermore, the high-fat (low-carbohydrate) diet led to the fastest weight regain. It appears that the CIM model does not agree with findings of the current study and does not explain effects of dietary macronutrients on adiposity in mice [32].

Consistent with our findings, previous research has also shown that high-fat diets promote adiposity in mice with unrestricted food intake. A study of 29 different diets with varying macronutrient compositions found that high-fat diets stimulate greater energy intake due to the hedonic effects of dietary fat in the hypothalamus [33]. Interestingly, we observed that mice on the high-fat diet had higher energy intake despite higher leptin and similar ghrelin levels compared to other diets during the refeeding phase of our study. This increased leptin-to-ghrelin ratio, considered as satiety and hunger signals, respectively, conflicts with the observed increased energy intake and sustained weight gain in mice on a high-fat diet. However, leptin is primarily produced in fat cells and reflects body fat content [34]. Thus, its increase probably serves as a marker of adiposity rather than satiety in this study. This warrants a search for other factors that control food intake during the refeeding phase after weight loss.

Our study also investigated whether a high-protein diet can reduce weight gain during the refeeding after weight loss. The protein leverage theory proposes that mammals prioritize protein intake and adjust their food consumption accordingly [35]. High-protein foods are hypothesized to be more satiating and reduce overeating when consumed in ad libitum conditions. Our study found no significant advantage of the high-protein diet for weight maintenance over the lower-protein diets. We compared the high-protein diet (35% kcal) with moderate carbohydrates and fat to two diets with lower protein content (20% kcal), i.e., high-carbohydrate or high-fat diets. There were no significant differences in body composition or hormone levels between the high-protein diet and high-carbohydrate diet with significantly lower protein content. A limitation of our comparison is that this high-carbohydrate diet already had a substantial protein content, which may limit the potential benefits of further protein intake. Future studies may consider lower-protein diets to better investigate the protein leverage concept. Mixed effects of protein on energy intake and body mass regulation have also been reported in other mouse studies. Although some studies support the notion of protein leverage in mice [36], others have shown it to be non-essential. In fact, a recent study has shown that, when fat content of the diet is not high, food intake in mice is more determined with energy rather than protein targets [33].

The major limitation of our study is the lack of additional methods for assessing body composition at the whole-body level (e.g., DXA scanning or others). We recognize that partial estimation of muscle and fat mass by dissecting a small fraction of individual muscles and fats may not reveal the full picture of changes in body composition. Also, when testing and challenging the above-mentioned CIM for obesity, we did not measure insulin concentrations due to insufficient blood samples. In addition to leptin and ghrelin data, it would also have been useful to examine the levels of certain gut-derived hormone incretins (e.g., GIP, GLP-1) related to glucose metabolism and mediating satiety [37].

## 5. Conclusions

Our findings highlight that the effects of macronutrient allocation in the diet on body composition in aged mice are dependent on energy balance. During isocaloric energy restriction, the dietary macronutrient composition has a relatively smaller impact on weight and fat loss than caloric restriction itself. However, when mice are exposed to ad libitum refeeding, the high-fat and low-carbohydrate diet leads to the least favorable body composition outcomes, which induce rapid weight regain that overshoots initial weight levels before caloric restriction, despite increased fat oxidation and high serum leptin levels associated with this diet. Increase in body and fat mass with this diet is explained with increased ad libitum energy intake.

## Data Availability

The data that support the findings of this study are available from the corresponding author upon reasonable request.
